# Review of "Adaptation to life at high salt concentrations in Archaea, Bacteria, and Eukarya" Edited by Nina Gunde-Cimerman, Aharon Oren, and Ana Plemenitaš

**DOI:** 10.1186/1746-1448-1-6

**Published:** 2005-08-17

**Authors:** Christopher Rensing

**Affiliations:** 1 Department of Soil, Water, and Environmental Science, University of Arizona, Tucson, AZ 85721, USA

## Abstract

The diversity of hypersaline environments and the physiology of representative organisms are only beginning to be understood. Recent progress in this area is documented in "Adaptation to life at high salt concentrations in Archaea, Bacteria, and Eukarya" – eds. Nina Gunde-Cimerman, Aharon Oren and Ana Plemenitas. The 34 chapters successfully paint a fascinating emerging picture of these environments and the microorganisms inhabiting them.

## Book details

Adaption to life at high salt concentrations in Archaea, Bacteria and Eukarya. *Series: Cellular Origin, Life in Extreme Habitatsand Astrobiology, Vol 9* 2005. Hardcover ISBN: 1-4020-3632-9

## 

**Figure 1:**
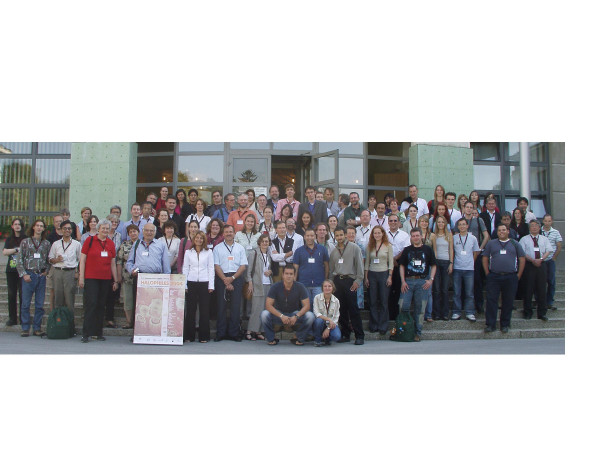
Participants of the recent international conference "Halophiles 2004" in Ljubljana.

Starting a new on-line journal such as Saline Systems presents the opportunity for editors and potential readers to familiarize themselves with recent ongoing research in this rather broad field. What better way to begin than to summarize new results and developments presented at a well-attended and received scientific meeting? This is the idea behind "Adaptations to life at high salt concentrations in Archaea, Bacteria and Eukarya" which explores the many-fold aspects of life under these extreme conditions. The book was initiated at an international conference "Halophiles 2004" in Ljubljana, Slovenia, in September 2004 organized by Nina Gunde-Cimerman and Ana Plemenitas, a conference which has been held roughly every three years since 1978 (Fig. 1). The first conference was held in Rehovot/Israel and organized by Roy Caplan and Margaret Ginzburg. A comparison of topics covered by conferences on halophilic microorganisms twenty years ago and more recently reflects how rapidly this field has expanded. The 34 chapters capture the current breadth of the field and illustrate the versatility of these microorganisms. Halophilic environments extend from the Great Salt Lake to the Dead Sea but also to unexpected environments such as arctic regions, deep-sea hypersaline brines and possibly habitats in outer space. The study of extremophiles might also give clues for a better understanding of the origin of life here on earth but possibly also elsewhere hence the link to exobiology explored by Joseph Seckbach and Rocco Mancinelli in this volume.

If one were to give out last years Oscars for salt-lovers, "Two square off" would certainly take home the honor. In my and certainly also the media's opinion, the cultivation and initial characterization of Walsby's square archaeon was a major accomplishment. Henk Bolhuis' fun to read article certainly succeeds in capturing the fascination of this seemingly strange but widespread microbe. How did he finally manage to get a hold on '*Haloquadratum walsbyi*', nothing fancy, just using agarose instead of agar plates. Seemingly simple, it certainly hides many frustrating hours spent in the lab. Another successful strategy was employed by David Burns out of Mike Dyall-Smith's lab using dilution cultures in natural saltern water with just a few supplements. Interestingly, they both managed to observe large sheets of Walsby's square archaeon, measuring approximately 40 × 40 μm. If these structures constitute single cells, or readily dissolve into multiple square components under the conditions used by D. Burns, this will certainly be an area of future studies.

Studying microbial communities in the natural environment is inherently difficult due to technical and biological aspects. One advantage of microbial ecology studies in extreme environments is the relatively low diversity of the respective microbial communities. It is therefore not surprising that this invitation to study the diversity and composition in hypersaline environments was followed up by a number of scientists and the results from Great Salt Lake, the Dead Sea, salterns in California and Spain and ancient salt deposits summarized here. In addition, often neglected predators such as Haloviruses or Protozoa feeding on halophilic archaea and bacteria are not only described but also evaluated on their impact on microbial communities in three chapters.

The physiology of halophilic and halotolerant organisms is also covered in great detail and insight in this volume. How do they do this, how can any organism survive under these conditions? To survive and thrive in a hypersaline environment, microorganisms needed to develop mechanisms of osmoadaptation and salt tolerance. In nature, different solutions brought about by convergent evolution to a low water activity environment can be found in various organisms able to withstand high salt conditions. Not only haloarchaea, as initially thought, but also bacteria accumulate high internal concentrations of potassium and chloride. An important, environmentally relevant representative, *Salinibacter ruber*, is described in detail from a genomic and environmental perspective. Another surprising aspect of the physiology of some halophiles is their requirement for chloride. A more detailed account is given in an interesting chapter on chloride requirement in moderately halophilic bacteria by Volker Müller and Stefan Saum. Their initial hypothesis for the necessity of chloride is not as counterion as one might intuitively believe but as an inducer for a novel regulatory network. However, if that were true, wouldn't one expect mutants in the signaling pathway without a requirement for chloride?

Transcriptional regulation in haloarchaea was also described using gas vesicle synthesis as an example. We are clearly only at the beginning of understanding the largely unexplored interplay between bacterial-like repressors and an eukaryae-like transcription machinery. This, I believe, is one of the surprises from the recent or on-going genome sequencing efforts. Brian Berquist and coworkers provide a genome-wide COG-based inventory of regulators, basal transcriptional machinery, and DNA replication and repair systems in the two completely sequenced haloarchaea, *Halobacterium *sp. NRC-1 and *Haloarcula marismortui*. This showed nearly all the regulators to be bacterial, along with a few (laterally transferred?) components of the DNA replication and repair systems. These aspects of haloarchaeal genetics are likely to be the subject of significant interest in the near future.

Ana Plemenitas, Nina Gunde-Cimerman and their respective group members have to be commended for their Herculean effort setting the record straight on fungal response to low water activity and their contribution and impact in hypersaline environments in three extensive and well-researched chapters.

Is this a book worth having: by all means YES! Overall it contains a wealth of information that is really useful having as a resource. In addition, research strategies and thoughtful discussions are presented in chapters such as *Diversity of Microbial Communities: The Case of Solar Salterns *by Carlos Pedros-Alio. This methodology is often at least as important as mere facts and findings, especially for the hopefully intended target audience of graduate students and postdoctoral researchers.

